# Functional changes during inpatient pulmonary rehabilitation in Poland: an ICF-based analysis of functioning relevant to healthy aging and public health

**DOI:** 10.3389/fpubh.2026.1949234

**Published:** 2026-10-13

**Authors:** Karol Pawlak, Dorota Kiedik, Monika Raulinajtys-Grzybek, Piotr Rzymski, Andrzej Fal

**Affiliations:** 1 Polish Society of Public Health, Wrocław, Poland; 2 Department of Public Health, Division of Public Health, Wroclaw Medical University, Wrocław, Poland; 3 Department of Management Accounting, SGH Warsaw School of Economics, Warsaw, Poland; 4 Department of Environmental Medicine, Poznan University of Medical Sciences, Poznan, Poland; 5 Faculty of Medicine, Collegium Medicum, Cardinal Stefan Wyszyński University in Warsaw, Warsaw, Poland

**Keywords:** chronic respiratory disease, inpatient rehabilitation, International Classification of Functioning, disability and health, pulmonary rehabilitation, rehabilitation outcomes

## Abstract

**Introduction:**

The International Classification of Functioning, Disability and Health (ICF) provides a comprehensive framework for assessing rehabilitation outcomes beyond traditional physiological measures.

**Methods:**

This retrospective observational study used a longitudinal pre–post design and involved a secondary analysis of anonymized data collected during a national pilot implementation of an ICF-based functional assessment system in routine Polish rehabilitation services It aimed to characterize within-patient changes in functioning observed between admission and discharge among 100 adults undergoing inpatient pulmonary rehabilitation, with a mean age of 68.7 ± 10.8 years (with 75% in the ≥65 years group), at baseline and 2–3 weeks post-rehabilitation. Changes in ICF qualifiers for Body Functions (B), Activities and Participation (D), and Environmental Factors (E) were evaluated using paired comparisons.

**Results:**

At baseline, the greatest impairments were observed in energy and drive functions (b130), sleep functions (b134), pain (b280), exercise tolerance functions (b455), joint mobility (b710), muscle power (b730), muscle endurance (b740), changing basic body position (d410), maintaining a body position (d415), and walking (d450). Between admission and discharge, significant reductions in impairment severity were observed in several body function categories, including muscle power (median qualifier 3 → 1), muscle endurance (3 → 2), joint mobility (3 → 1), pain (b280), exercise tolerance (1 → 0), and sensations associated with cardiovascular and respiratory functions (2 → 1) (all adjusted *p* < 0.01). Significant reductions in activity limitations were also observed for changing basic body position (3 → 2), maintaining a body position (3 → 2), walking (2 → 1), transfers (severe/complete limitation: 17% → 4%), daily routines (severe/complete limitation: 36% → 19%), and multiple self-care activities multiple self-care activities, including washing (1 → 0), caring for body parts (1 → 0), toileting (severe/complete limitation: 2% → 0%), and dressing (1 → 0) (all adjusted *p* < 0.01). Environmental factors remained stable, with medications, assistive products and technologies and family support consistently identified as facilitators of functioning.

**Conclusion:**

These findings describe differences across several dimensions of ICF-rated functioning between admission and discharge during inpatient pulmonary rehabilitation. They suggest that routine ICF-based assessments may provide a useful multidimensional description of functioning. Future controlled studies could examine the extent to which these differences are attributable to rehabilitation, while dedicated validation studies could assess the responsiveness of the ICF-based assessment system and the clinical meaning of changes in qualifier scores.

## 1 Introduction

The International Classification of Functioning, Disability and Health (ICF), developed by the World Health Organization, offers a comprehensive biopsychosocial framework for describing health and disability (1, 2). Rather than focusing solely on disease processes and impairments, the ICF conceptualizes functioning as the result of dynamic interactions among health conditions, body functions and structures, activities, participation, and contextual factors, including environmental and personal influences. This broader perspective facilitates a more comprehensive evaluation of patient functioning by considering how health conditions affect daily activities, social engagement, and overall participation in life situations (3, 4).

This perspective is particularly relevant to aging and public health because maintaining functional ability, independence, participation, and quality of life is a central component of healthy aging. Evidence on rehabilitation outcomes in older persons indicates that ICF-based approaches can capture clinically and socially meaningful dimensions of functioning that extend beyond disease-specific measures (4, 5). For people living with chronic respiratory disease in later life, limitations in mobility, self-care, participation, and access to supportive environmental resources may influence independence and the need for health and social care. Accordingly, pulmonary rehabilitation can be considered not only a clinical intervention but also a public-health strategy that supports functional independence and healthy aging.

In rehabilitation practice, the ICF has emerged as an important framework for assessing outcomes that extend beyond traditional clinical indicators (5–7). Its standardized structure allows healthcare professionals to describe functioning consistently, improving interdisciplinary collaboration and supporting evidence-based decision-making. Furthermore, the ICF enables the assessment of multiple dimensions of health, including body functions, activities, participation, and environmental influences, thereby facilitating a more comprehensive evaluation of rehabilitation needs and outcomes (1, 8).

In pulmonary medicine, patient functioning is commonly evaluated using a range of clinical and functional assessment tools. Measures such as spirometry, including forced expiratory volume in one second (FEV1) and forced vital capacity (FVC), provide information on pulmonary function and disease severity (9). Exercise capacity is frequently assessed using the 6-min walk test, incremental shuttle walk test, or cardiopulmonary exercise testing. Dyspnea and symptom burden are commonly evaluated using instruments such as the Modified Medical Research Council Dyspnea Scale and the COPD Assessment Test, whereas health-related quality of life may be assessed using disease-specific questionnaires such as the St. George’s Respiratory Questionnaire (10). Although these instruments are clinically valuable and extensively validated, they typically focus on selected physiological parameters, symptoms, exercise performance, or quality-of-life dimensions. Consequently, multiple assessment tools are often required to capture the broad functional consequences of chronic respiratory disease. In contrast, the ICF provides a unified framework that integrates physiological impairment, activity limitations, participation restrictions, and contextual factors within a single conceptual model, enabling a more comprehensive evaluation of functioning across diverse pulmonary conditions (11, 12).

The implementation of the ICF in Poland has evolved gradually, with the most substantial progress occurring in medical rehabilitation. Following the adoption of the ICF by the World Health Organization in 2001, Polish healthcare institutions increasingly recognized its value for standardized assessment of functioning and rehabilitation outcomes. A significant milestone was the introduction of voluntary ICF-based reporting of selected functional assessment categories in rehabilitation services by the National Health Fund (NFZ) in 2019. Subsequently, the NFZ/POWR project “Development and implementation of a comprehensive functional assessment system in rehabilitation using international experiences and solutions” (POWR.04.03.00-00-0025/19) developed and tested standardized ICF-based assessment procedures for inpatient and outpatient rehabilitation. Although the ICF has not yet been uniformly integrated across the entire healthcare system, these initiatives have substantially expanded its application in rehabilitation practice through standardized functional profiles and digital assessment tools (13–16).

Pulmonary rehabilitation is a particularly relevant area for implementing the ICF framework (17, 18), especially in populations in later life in whom chronic respiratory disease may interact with age-related vulnerability, functional decline, and loss of independence. It is a comprehensive intervention designed for patients with chronic respiratory diseases, including chronic obstructive pulmonary disease (COPD), interstitial lung diseases, bronchiectasis, asthma, pulmonary hypertension, and selected post-infectious respiratory conditions (19–22). These disorders frequently result in a complex spectrum of impairments extending beyond pulmonary dysfunction, including exercise intolerance, fatigue, muscle weakness, limitations in daily activities, reduced social participation, and diminished quality of life (23–25). Consequently, pulmonary rehabilitation requires a multidimensional and patient-centered approach that addresses not only respiratory symptoms but also the broader functional consequences of chronic disease (26). Therefore, the ICF framework may provide a valuable structure for capturing these diverse dimensions of functioning and for monitoring rehabilitation-related changes that are relevant to maintaining independence and healthy aging.

Despite the well-established clinical benefits of pulmonary rehabilitation, outcome evaluation remains largely focused on physiological measures, exercise performance, symptom scores, and quality-of-life indicators (18, 22). Compared with comprehensive assessments of functioning, less attention has been devoted to those that simultaneously address body functions, activities, participation, and environmental influences (27). The ICF framework offers an opportunity to bridge this gap by providing a standardized language and measurement structure capable of capturing multidimensional rehabilitation outcomes. Nevertheless, there remains a need for large-scale, real-world analyses examining the applicability of ICF-based assessments in pulmonary rehabilitation settings.

Therefore, this study aimed to describe and quantify within-patient changes in functioning among adults undergoing inpatient pulmonary rehabilitation in Poland, in a cohort of predominantly older adults, using a standardized ICF-based assessment system implemented in Polish rehabilitation services. The primary endpoint was the change in ICF qualifier scores between baseline and final assessment for predefined ICF categories within the domains of body functions, activities, and participation. Secondary endpoints included changes in environmental factor qualifiers, analyzed separately as barriers and facilitators, and changes in the proportion of patients with severe or complete problems in selected ICF categories. The public-health relevance of the study lies in evaluating functional independence and contextual factors that may influence the ability of adults in later life to maintain participation and autonomy after rehabilitation. Previous Polish studies have supported the implementation of the ICF in rehabilitation by developing Polish rating guides, validating ICF-based assessment tools, evaluating selected ICF Core Sets, and conducting system-level implementation projects (13–16). The present study builds upon this work but addresses a different research question. Rather than developing or validating an ICF instrument, it applies a standardized ICF-based assessment system to a real-world cohort of patients undergoing inpatient pulmonary rehabilitation. Its distinct contribution lies in the paired baseline-to-follow-up analysis of multidimensional functional change across body functions, activities, and participation, as well as environmental factors, in a heterogeneous pulmonary rehabilitation population. Thus, the novelty of this study lies not in creating a new ICF tool but in the profile-specific analysis of functional outcomes and their implications for maintaining independence in a population with substantial representation of later-life adults.

## 2 Materials and methods

### 2.1 Study design and population

This study was a secondary analysis of anonymized data collected during the National Health Fund (NFZ)/POWR pilot implementation of an International Classification of Functioning, Disability and Health (ICF)-based functional assessment system in rehabilitation services in Poland. The national pilot was conducted between March and October 2023 and included 1,797 patients across five rehabilitation profiles. The present single-center analysis included all 100 adults assigned to the inpatient pulmonary rehabilitation profile at Provincial Rehabilitation Hospital Dr. S. Jasiński in Zakopane, Poland. The cohort had a mean age of 68.7 ± 10.8 years, providing a clear later-life and healthy-aging context for the analysis. Each patient contributed one inpatient rehabilitation episode, with assessments at admission and discharge. Inpatient rehabilitation episodes lasted 2–3 weeks, but the secondary dataset did not provide information on the clinical or organizational factors determining their exact duration. The dataset included age, sex, assessment dates, rehabilitation profile, professional category of the assessor, ICF qualifier scores, and ICD-9 medical procedure groups. Detailed clinical information, including specific respiratory diagnoses, disease severity, use of oxygen therapy, and detailed characteristics of the rehabilitation program such as the number of sessions, training intensity, and frequency, was not available in the secondary dataset. Because the secondary dataset did not provide the detailed age-stratified clinical variables needed for age-specific subgroup analyses, the study focuses on the overall cohort and does not make age-group-specific causal inferences.

The present analysis used a longitudinal pre–post design. Functional assessments were performed at the beginning and at the completion of the rehabilitation episode. Changes in ICF qualifier scores between baseline and follow-up assessments were analyzed to evaluate functional changes in functioning observed over the course of the rehabilitation episode. This study involved a secondary analysis of irreversibly anonymized data collected during the implementation of an ICF-based functional assessment documentation system in routine inpatient rehabilitation services in Poland. The dataset comprised only non-identifiable records extracted from an implementation monitoring system. The authors had no involvement in patient recruitment or clinical care and had no access to personal identifiers or any means of re-identifying participants. Admission and discharge assessments were linked using a technical identifier created solely for data management purposes. The underlying implementation project evaluated a standardized approach to documenting functioning in routine rehabilitation practice and did not introduce experimental interventions or alter clinical care. Functional assessments were performed by healthcare professionals as part of standard rehabilitation procedures. This study involved no direct patient contact, additional data collection, or procedures beyond routine care. Because the analysis was based exclusively on irreversibly anonymized data generated during routine healthcare delivery, ethics committee or institutional review board approval was not required. Separate informed consent for this secondary analysis was not required; any consent related to hospitalization, rehabilitation, and routine clinical documentation was obtained by participating healthcare providers in accordance with applicable regulations.

### 2.2 Functional assessment with ICF

Functional assessment was performed using the ICF, covering body functions (B), activities and participation (D), and environmental factors (E). Assessments were entered into a dedicated electronic documentation system developed within the NFZ/POWR project. ICF coding and qualifier assignment were performed by trained healthcare professionals as part of routine clinical practice. The ICF categories included in the assessment profiles were predefined by a multidisciplinary expert team, and all members of the pilot assessment teams underwent mandatory project-specific training covering the ICF, categorical profiles, assessment methods, and use of the electronic documentation system. In the inpatient setting, functional assessment was team-based and involved at least a physician, physiotherapist, and nurse; the available project documentation does not indicate whether the same team member rated a given ICF category at admission and discharge.

For body functions and activities and participation, ICF qualifiers were scored on an ordinal problem scale: 0 = no problem, 1 = mild problem, 2 = moderate problem, 3 = severe problem, and 4 = complete problem. The values 8 and 9 denoted “not specified” and “not applicable,” respectively. Environmental factors were evaluated in two directions. When an environmental factor limited functioning, it was scored as a barrier on the negative 0–4 scale. When it supported functioning, it was scored as a facilitator on a positive scale: 0 = no facilitator, +1 = mild facilitator, +2 = moderate facilitator, +3 = substantial facilitator, and +4 = complete facilitator. Qualifier assignment was based on structured assessment forms developed within the NFZ/POWR project. These forms included questionnaires, clinical measurements, and predefined conversion algorithms linking assessment results to ICF qualifiers. Thus, the numerical ICF qualifiers used in the analysis were not free-text subjective ratings but standardized outputs of the pilot functional assessment documentation system. Each patient underwent two assessments: a baseline assessment at admission and a follow-up assessment at discharge. The resulting ICF qualifiers constituted the primary study outcomes.

### 2.3 Statistical analyses

Before analysis, records were deduplicated at the patient–assessment–ICF category level. Duplicate records containing identical qualifier values for the same patient, assessment time point, and ICF category were collapsed into a single observation. Missing values and qualifiers 8 (“not specified”) and 9 (“not applicable”) were treated as non-evaluable and excluded from the calculation of descriptive statistics and paired comparisons. Paired analyses were restricted to patients with valid qualifier values at both admission and discharge for the relevant ICF category. Descriptive statistics were used to summarize demographic and clinical characteristics. Ordinal variables were reported as medians and interquartile ranges (IQR), whereas categorical variables were presented as frequencies and percentages. Changes between baseline and follow-up ICF qualifiers were evaluated using the Wilcoxon signed-rank test. Because ICF qualifiers represent a restricted ordinal scale with a substantial number of tied values, the magnitude of change was described using complementary distribution-based measures, including admission and discharge medians with interquartile ranges and the proportions of patients with severe or complete problems. Confidence intervals for median within-patient differences were not calculated because the highly discrete distribution of change scores limits their interpretability. Similarly, standardized rank-based effect sizes were not used as measures of clinical magnitude because clinically meaningful thresholds for such effect sizes have not been established for individual ICF qualifier categories. *p* values were adjusted for multiple testing using the Benjamini–Hochberg procedure, applied separately within each ICF domain to control the false discovery rate among conceptually related comparisons. Statistical significance was defined as *p* < 0.05.

## 3 Results

### 3.1 Main characteristics of the studied group

The pulmonary rehabilitation cohort consisted of 100 adults with a mean±SD age of 68.7 ± 10.8 years (min-max 35–89 years). Seventy-five participants (75%) were aged 65 years or older, indicating that the study population was predominantly situated in later adulthood. Women accounted for 57% of the cohort and men for 43%. The interval between baseline and final assessment was 2 weeks in 5 patients (5%) and 3 weeks in 95 patients (95%), reflecting the typical duration of inpatient pulmonary rehabilitation. Baseline and discharge assessments included category-level entries made by physicians, physiotherapists, and nurses.

### 3.2 Changes in body functions

Detailed results for individual ICF categories within the Body Functions (B) domain are presented in Table 1. At baseline, the most pronounced impairments were observed in energy and drive functions (b130), sleep functions (b134), pain (b280), exercise tolerance functions (b455), joint mobility functions (b710), muscle power functions (b730), and muscle endurance functions (b740), with a substantial proportion of patients reporting severe limitations. Between the initial and final assessments, significant changes were observed across most body function categories. The largest reductions in impairment severity were noted for musculoskeletal functions, particularly muscle power (b730; median qualifier 3 → 1, adjusted *p* < 0.0001), muscle endurance (b740; 3 → 2, adjusted *p* < 0.0001), and joint mobility (b710; 3 → 1, adjusted *p* < 0.0001). Marked decreases in impairment severity were also observed for pain (b280; 3 → 2), exercise tolerance functions (b455; 1 → 0), sensations associated with cardiovascular and respiratory functions (b460; 2 → 1). Lower impairment qualifiers were also recorded at discharge for respiratory muscle functions (b445) and attention functions (b140), although baseline impairment in these areas was less pronounced. In contrast, emotional functions (b152), weight maintenance functions (b530), and selected respiratory and cardiac functions showed little or no change in the reported qualifiers.

**Table 1:** Body functions (ICF domain B) before and after pulmonary rehabilitation.

ICF code	Domain	*n*	Before rehabilitation	After rehabilitation	Before rehabilitation	After rehabilitation	Adjusted *p*-value
			Median (Q1–Q3)		% severe (score 3 + 4)		
b110	Consciousness functions	100	0 (0–0)	0 (0–0)	0.0	0.0	–
b114	Orientation functions	100	0 (0–0)	0 (0–0)	2.0	2.0	–
b130	Energy and drive functions	100	3 (2–3)	2 (1–3)	66.0	39.0	<0.0001
b134	Sleep functions	100	3 (2–3)	2 (2–3)	59.0	45.0	<0.0001
b140	Attention functions	100	1 (0–2)	0 (0–1.5)	14.0	10.0	0.005
b152	Emotional functions	100	1 (1–2)	1 (1–2)	8.0	9.0	0.139
b280	Sensation of pain	100	3 (3–3)	2 (1–3)	80.0	41.0	<0.0001
b4100	Heart rate functions	100	0 (0–0)	0 (0–0)	0.0	0.0	0.82
b4101	Heart rhythm functions	100	1 (0–2)	0 (0–1)	6.0	3.0	<0.0001
b4102	Heart contraction force functions	100	0 (0–0)	0 (0–0)	5.0	5.0	0.629
b4103	Blood supply to the heart functions	100	1 (0–2)	0 (0–1)	6.0	0.0	<0.0001
b4150	Artery functions	100	0 (0–0)	0 (0–0)	1.0	2.0	–
b4200	Increased blood pressure functions	100	2 (1–3)	1 (0–2.5)	34.0	25.0	0.008
b4201	Decreased blood pressure functions	100	0 (0–0)	0 (0–0)	11.0	2.0	0.016
b4202	Maintaining blood pressure functions	100	0 (0–0)	0 (0–0)	20.0	4.0	0.0004
b4400	Respiration rate functions	100	0 (0–0)	0 (0–0)	4.0	2.0	0.013
b4401	Respiratory rhythm functions	100	0 (0–0)	0 (0–0)	0.0	0.0	–
b4402	Depth of respiration functions	92	2 (1–3)	2 (1–3)	26.0	29.0	0.266
b445	Respiratory muscle functions	100	0 (0–2)	0 (0–1)	13.0	7.0	0.002
b450	Additional respiratory functions	96	2 (2–3)	2 (2–3)	49.0	40.0	0.252
b455	Exercise tolerance functions	100	1 (0–1)	0 (0–1)	8.0	8.0	<0.0001
b460	Sensations associated with cardiovascular and respiratory functions	100	2 (1–3)	1 (1–2)	35.0	8.0	<0.0001
b530	Weight maintenance functions	100	1 (0–1.5)	1 (0–1)	9.0	9.0	1.000
b620	Urination functions	100	1 (0–2)	1 (0–1)	4.0	2.0	0.043
b710	Mobility of joint functions	100	3 (2–3)	1 (1–2)	51.0	13.0	<0.0001
b730	Muscle power functions	65	3 (1–3)	1 (0–1)	60.0	1.5	<0.0001
b740	Muscle endurance functions	100	3 (3–3)	2 (2–3)	78.0	33.0	<0.0001

ICF qualifiers were scored as follows: 0 = no problem, 1 = mild problem, 2 = moderate problem, 3 = severe problem, and 4 = complete problem. “Severe/complete problem” denotes the proportion of patients with qualifier 3 or 4.

### 3.3 Changes in activities and participation

Detailed results for individual ICF categories within the Activities and Participation domain (D) are presented in Table 2. At baseline, the most pronounced activity limitations were observed for changing basic body position (d410), maintaining a body position (d415), walking (d450), and carrying out daily routines (d230). Admission-to-discharge differences were observed across most activity-related categories. The largest changes were observed for changing basic body position (d410; median qualifier 3 → 1), maintaining a body position (d415; 3 → 2), and walking (d450; 2 → 1) (all adjusted *p* < 0.0001). Significant changes were also observed for transfers (severe/complete limitation: 17% → 4%), daily routines (36% → 19%), and several self-care activities, including washing oneself (median qualifier 1 → 0), caring for body parts (1 → 0), and dressing (1 → 0) (all adjusted *p* < 0.01). Although statistically significant changes were observed in handling stress and other psychological demands (d240) and basic interpersonal interactions (d710), baseline impairment in these domains was generally low. In contrast, little or no change was observed for eating (d550) and moving around using equipment (d465), largely reflecting the minimal baseline limitations reported in these activities.

**Table 2:** Activities and participation (ICF domain D) before and after pulmonary rehabilitation.

ICF code	Domain	*n*	Before rehabilitation	After rehabilitation	Before rehabilitation	After rehabilitation	Adjusted *p*-value
			Median (Q1–Q3)		% severe (score 3 + 4)		
d230	Carrying out the daily routine	100	2 (0–3)	2 (0–2)	36.0	19.0	<0.0001
d240	Handling stress and other psychological demands	100	0 (0–1)	0 (0–1)	0.0	0.0	0.0002
d410	Changing basic body position	100	3 (2–3)	1 (0–3)	51.0	26.0	<0.0001
d415	Maintaining a body position	100	3 (2–3)	2 (1–3)	71.0	32.0	<0.0001
d420	Transferring oneself	100	0 (0–2)	0 (0–0)	17.0	4.0	<0.0001
d450	Walking	100	2 (2–3)	1 (1–2)	44.0	15.0	<0.0001
d465	Moving around using equipment	100	0 (0–0)	0 (0–0)	1.0	0.0	0.18
d510	Washing oneself	100	1 (0–2)	0 (0–1)	12.0	6.0	0.002
d520	Caring for body parts	100	1 (0–2)	0 (0–1)	12.0	5.0	0.002
d530	Toileting	100	0 (0–0)	0 (0–0)	2.0	0.0	0.019
d540	Dressing	100	1 (0–2)	0 (0–1)	13.0	11.0	0.002
d550	Eating	100	0 (0–0)	0 (0–0)	0.0	1.0	0.167
d570	Looking after one’s health	100	1 (0.5–2)	1 (0–1)	5.0	1.0	0.0002
d710	Basic interpersonal interactions	100	0 (0–0)	0 (0–0)	1.0	0.0	0.010

ICF qualifiers were scored as follows: 0 = no problem, 1 = mild problem, 2 = moderate problem, 3 = severe problem, and 4 = complete problem. “Severe/complete problem” denotes the proportion of patients with qualifier 3 or 4.

### 3.4 Changes in environmental factors

Environmental factors (ICF domain E) were generally stable throughout the rehabilitation period. No statistically significant changes were observed for environmental barriers or facilitators related to medications, assistive technologies, architectural features, family support, or support from caregivers and personal assistants (Figure 1). Baseline assessments indicated that medications (e1101), assistive products and technologies (e1151), and family support (e310) were frequently perceived as facilitators of functioning, and these positive perceptions remained largely unchanged at discharge.

**Figure 1:**
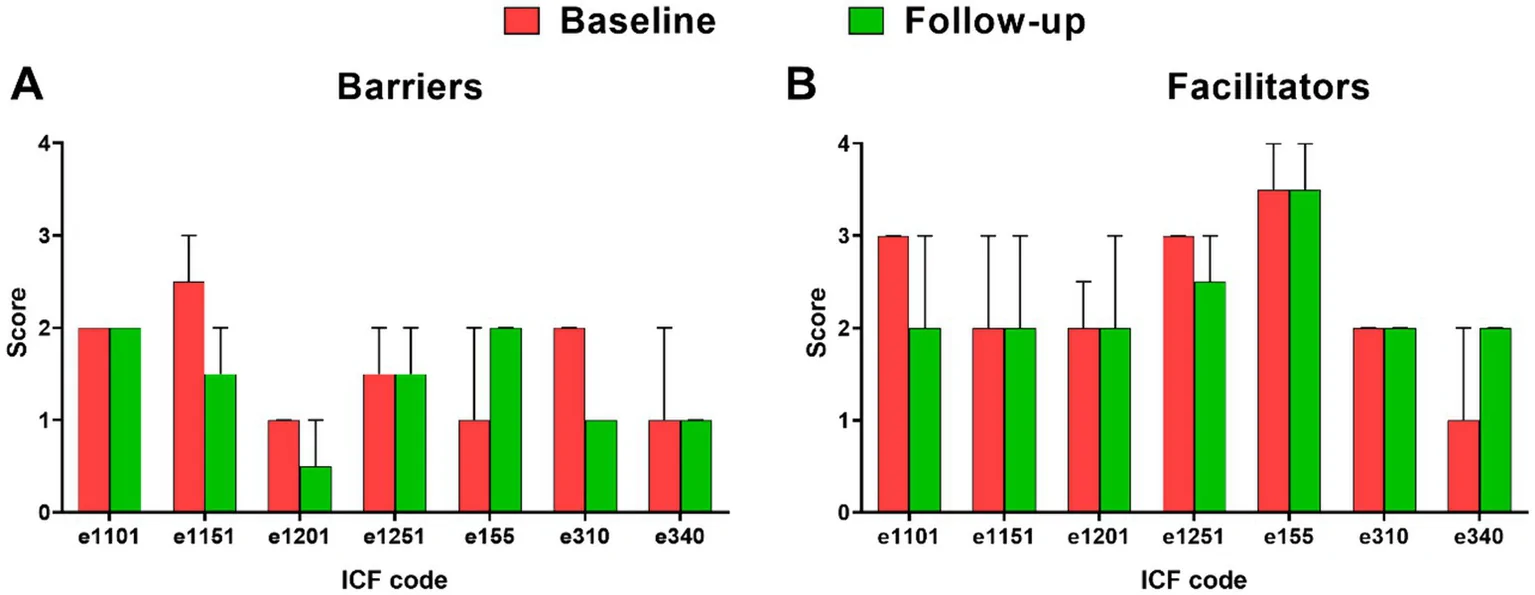
Environmental factors (ICF domain E) before and after pulmonary rehabilitation. In the barrier scale **(A)**, 0 = no barrier, 1 = mild barrier, 2 = moderate barrier, 3 = severe barrier, and 4 = complete barrier. In the facilitator scale **(B)**, 0 = no facilitator, +1 = mild facilitator, +2 = moderate facilitator, +3 = substantial facilitator, and +4 = complete facilitator. Bars represent medians, while whiskers represent interquartile range. No significant differences between baseline and follow-up measurements were detected (*p* > 0.05).

## 4 Discussion

This study provides a real-world description of changes in functioning among adults undergoing inpatient pulmonary rehabilitation in Poland using a standardized ICF-based assessment system. The cohort consisted of predominantly older adults, making the findings particularly relevant to functional independence and healthy aging. To our knowledge, this is one of the largest analyses of longitudinal ICF-based functional assessments conducted in a pulmonary rehabilitation setting in Poland. The findings show that significant changes between admission and discharge assessments were observed across multiple dimensions of functioning, particularly within body functions, activities, and participation. Importantly, the study extends the assessment of functioning during pulmonary rehabilitation beyond traditional physiological and exercise-based outcomes by capturing a broad spectrum of functioning, including mobility, self-care, participation, and environmental factors. These dimensions are directly relevant to the ability of people in later life to maintain autonomy and participation and therefore provide a public-health perspective on functional assessment in rehabilitation. The observations illustrate how a routine ICF profile can describe several dimensions of functioning at admission and discharge (11, 18). However, whether the observed qualifier differences accurately reflect change in patients’ long-term functioning requires a further dedicated assessment of responsiveness.

Within the Body Functions domain, the greatest baseline impairments were observed in categories related to fatigue and vitality, sleep, pain, exercise tolerance, muscle strength, muscle endurance, and joint mobility. These findings are consistent with the multidimensional burden of chronic respiratory diseases, which frequently extend beyond pulmonary dysfunction and involve systemic manifestations such as skeletal muscle deconditioning, reduced physical capacity, fatigue, and symptom burden (28, 29). Particularly notable were the reductions in impairment severity observed for muscle power, muscle endurance, and joint mobility. These categories represent core components of physical functioning and are closely linked to exercise capacity and overall independence. Substantial changes were also observed in pain, exercise tolerance, and sensations associated with cardiovascular and respiratory functions. Such observations are consistent with previous reports indicating that individuals undergoing pulmonary rehabilitation often experience improvements in symptom burden and functional performance (29, 30). For exercise tolerance (b455), the median qualifier decreased from 1 to 0, although the proportion of patients with severe or complete problems remained 8% at both assessments. This suggests that the observed difference primarily involved patients in the lower qualifier categories.

In contrast, relatively little change was observed in emotional functions, weight maintenance, and several cardiac and respiratory categories. This pattern may reflect the fact that some aspects of functioning are less amenable to short-term change, particularly during a rehabilitation episode typically lasting only two to three weeks, a period that may be insufficient to produce measurable changes in various aspects of mental well-being and mental health of patients (31, 32). Furthermore, several categories demonstrated limited baseline impairment, reducing the potential for measurable improvement. Emotional functioning may also be influenced by broader psychosocial factors that are not routinely addressed within standard rehabilitation programs (33, 34).

The pattern observed in the Activities and Participation domain complements the findings for body functions. The largest changes were recorded in mobility-related categories, including changing and maintaining body position, transfers, and walking. These activities are fundamental determinants of independence and frequently represent major challenges for individuals with chronic respiratory diseases due to dyspnea, reduced exercise tolerance, muscle weakness, and physical deconditioning (28). Reductions in limitations were also observed in self-care activities such as washing, dressing, and personal care, as well as in carrying out daily routines and looking after one’s health. The simultaneous observation of changes in mobility and self-care domains suggests a broader shift toward improved functional independence over the course of the rehabilitation episode. In contrast, little change was observed for eating and moving around using equipment. This likely reflects the low prevalence of baseline limitations in these categories, producing a ceiling effect that limited the possibility of detecting meaningful change. Similarly, although statistically significant changes were observed in stress management and interpersonal interactions, baseline impairment in these domains was generally low. For handling stress and other psychological demands, the significant paired result despite an unchanged median and severe/complete-problem proportion may likewise reflect relatively small but systematic shifts within the lower qualifier categories. Thus, statistical significance in this domain should not be interpreted as indicating a large change in overall impairment severity, and its practical relevance should be considered cautiously.

Environmental factors exhibited substantially greater stability than body functions or activity-related categories. Medications, assistive technologies, family support, and support from caregivers were commonly identified as facilitators of functioning at baseline and generally retained their perceived importance at discharge. Similarly, environmental barriers were infrequently reported and showed little change over time. This pattern is not unexpected because many environmental factors reflect relatively stable contextual characteristics that are unlikely to change during a short inpatient rehabilitation episode. Access to medications, assistive products, architectural features of the home environment, and social support networks are often determined by circumstances extending beyond the rehabilitation setting itself (35, 36). Therefore, stability of environmental factors observed in this study may indicate that the principal changes recorded between assessments occurred within the individual’s functioning rather than within the surrounding environment. At the same time, the frequent identification of medications, assistive technologies, and family support as facilitators underscores the importance of contextual resources in shaping the lived experience of chronic respiratory disease and supports the inclusion of environmental factors within comprehensive rehabilitation assessments (12).

The functional changes observed in mobility, transfers, walking, self-care, and daily routines are relevant beyond the immediate clinical episode because these domains contribute to independence and participation in later life. A standardized ICF profile can therefore provide information that is meaningful for rehabilitation planning, continuity of care, and identification of functional needs that may require support after discharge. The relative stability of environmental factors also suggests that changes in functioning observed during rehabilitation should be interpreted within the wider social and service context, including access to medications, assistive products, family support, and caregiver assistance. In an aging population, integrating these functional and contextual dimensions into routine rehabilitation assessment may help health systems move from disease-centered outcome monitoring toward approaches that better support healthy aging, independence, and equitable access to supportive services. These implications should be considered as potential applications of the findings rather than as effects directly demonstrated by this observational study.

The findings should be interpreted in light of several limitations. First, the study was based on a retrospective secondary analysis of routinely collected clinical data, and therefore, the observed changes cannot be interpreted as evidence of causal effects of pulmonary rehabilitation. The absence of a control group prevents distinction between changes associated with rehabilitation, natural recovery, regression to the mean, or other concurrent influences. Second, the study population comprised a heterogeneous group of patients undergoing pulmonary rehabilitation, and information on specific respiratory diagnoses, disease severity, comorbidities, treatment regimens and rehabilitation program (the number of sessions and the frequency and intensity of training) was unavailable. Consequently, it was not possible to stratify analyses by underlying disease characteristics or rehabilitation exposure, or to identify subgroups with distinct patterns of functional change. This limited clinical characterization should also be considered when interpreting the generalizability of the findings to specific diagnostic or disease-severity groups. Third, although the cohort had a mean age of 68.7 years, detailed age-stratified analyses were not possible; therefore, the findings should not be generalized to all older adults or interpreted as demonstrating age-specific effects. Fourth, the follow-up period was limited to the duration of inpatient rehabilitation, precluding assessment of the persistence of observed changes after discharge. Fifth, although the ICF-based assessment system was standardized and implemented through structured documentation procedures, ICF qualifiers remain ordinal measures and may be influenced by individual assessors’ interpretations. Moreover, because the available project documentation did not establish that the same professional performed corresponding admission and discharge ratings, potential between-evaluator variability cannot be excluded. Finally, the study was conducted within the Polish healthcare system and within the framework of a national implementation project, which may limit the generalizability of the findings to other healthcare settings. Despite these limitations, the study provides a real-world description of admission-to-discharge differences in ICF qualifiers across several dimensions of functioning. The findings support further investigation of the assessment system’s measurement properties and potential relevance to rehabilitation planning.

## 5 Conclusion

In this real-world cohort of predominantly older adults undergoing inpatient pulmonary rehabilitation, ICF-based assessments recorded admission-to-discharge differences in multiple dimensions of functioning between admission and discharge. The most pronounced changes were observed in body functions related to musculoskeletal performance, symptom burden, and exercise tolerance, accompanied by reductions in activity limitations affecting mobility, self-care, and daily routines. In contrast, environmental factors remained largely stable throughout the rehabilitation episode, suggesting relative stability of these contextual influences over the short observation period. The findings of this retrospective secondary analysis suggest that ICF-based assessments may be useful for describing functioning across multiple domains during inpatient rehabilitation. Although the study does not establish age-specific or causal effects, its focus on multidimensional functioning in a cohort with a mean age in later adulthood provides a relevant basis for linking functional assessment in rehabilitation with broader goals of autonomy, participation, and healthy aging.

## Data Availability

The raw data supporting the conclusions of this article will be made available by the authors, without undue reservation.
